# Fabrication of Crystalline Microresonators of High Quality Factors with a Controllable Wedge Angle on Lithium Niobate on Insulator

**DOI:** 10.3390/nano9091218

**Published:** 2019-08-29

**Authors:** Jianhao Zhang, Zhiwei Fang, Jintian Lin, Junxia Zhou, Min Wang, Rongbo Wu, Renhong Gao, Ya Cheng

**Affiliations:** 1 State Key Laboratory of High Field Laser Physics, Shanghai Institute of Optics and Fine Mechanics, Chinese Academy of Sciences, Shanghai 201800, China; 2 Center of Materials Science and Optoelectronics Engineering, University of Chinese Academy of Sciences, Beijing 100049, China; 3 State Key Laboratory of Precision Spectroscopy, East China Normal University, Shanghai 200062, China; 4 The Extreme Optoelectromechanics Laboratory, School of Physics and Materials Science, East China Normal University, Shanghai 200241, China; 5 Collaborative Innovation Center of Extreme Optics, Shanxi University, Taiyuan 030006, China

**Keywords:** lithium niobate microdisk resonator, controllable wedge angle, high quality factors, chemo-mechanical polish

## Abstract

We report the fabrication of crystalline microresonators of high quality (Q) factors with a controllable wedge angle on lithium niobate on insulator (LNOI). Our technique relies on a femtosecond laser assisted chemo-mechanical polish, which allows us to achieve ultrahigh surface smoothness as critically demanded by high Q microresonator applications. We show that by refining the polish parameters, Q factors as high as 4.7 × 10^7^ can be obtained and the wedge angle of the LNOI can be continuously tuned from 9° to 51°.

## 1. Introduction

Whispering gallery microresonators (WGM) are miniaturized optical cavities where light waves can travel along the circular periphery with extremely low propagation losses by means of total internal reflection at the glass–air interface. So far, WGMs have been realized in various kinds of transparent materials such as liquids, polymers, glasses, semiconductors, and dielectric crystals [1,2,3]. The physical and/or optical functionalities demonstrated with WGMs include lasing, filtering, nonlinear wavelength conversion, optomechanics, and cavity electrodynamics, to name only a few [4,5,6,7,8,9]. Currently, it is of high interest to realize on-chip integration of the WGMs with other photonic micro- and nanostructures. The material platforms suitable for photonic integration application include traditional semiconductors for their fabrication compatibility with the complementary metal oxide semiconductor(CMOS) approach and the emerging lithium niobate on insulator (LNOI), thanks to the rapid development of innovative solutions to produce high quality LNOI photonic structures [10,11,12,13,14,15].

Very recently, we have demonstrated the fabrication of high Q LNOI microdisk resonators using chemo-mechanical polish lithography [16,17,18]. The Q factor was measured as 1.46 × 10^7^ at a wavelength of around 773 nm. As a typical feature of chemo-mechanical polish, the fabricated microdisk showed an extended wedge at the rim of a wedge angle of ~9.5°. High Q microresonators have been used to demonstrate electric tunable optomechanics [19]. Nevertheless, for most WGM applications, it is always desirable to improve the Q factor and to have the capability of controlling the dispersion property with a tunable wedge angle. This provides a strong incentive for us to carry out systematic investigations into the optimizations of the Q factor and wedge angle by refining the fabrication parameters, which will be shown in detail below.

## 2. Experimental Details

In our investigation, the LNOI microdisks with variable diameters and wedge angles were produced on a commercially available X-cut LNOI wafer (NANOLN, Jinan Jingzheng Electronics Co. Ltd. Jinan, China). The lithium niobate (LN) thin film with a thickness of 900 nm was bonded to a 2 μm-thick SiO_2_ layer supported by a 500-μm-thick LN substrate.

**Figure 1:**
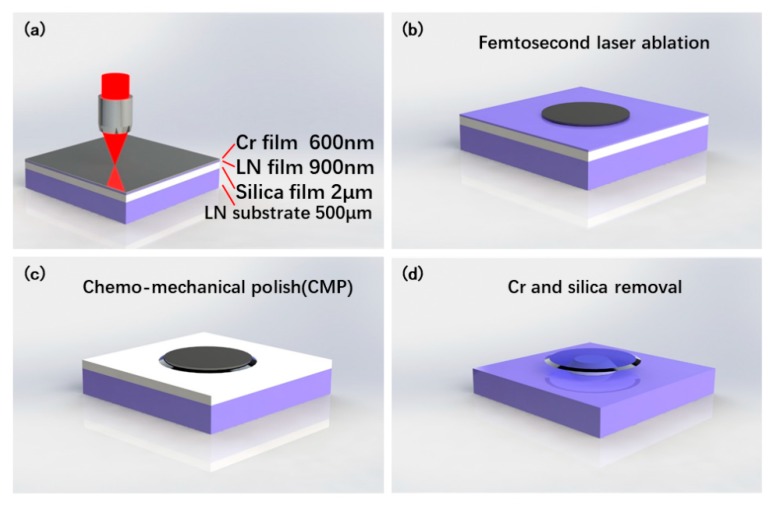
(**a**–**d**) Flowchart of fabricating an on-chip lithium niobate microdisk resonator.

As shown in Figure 1, the fabrication process includes four steps as briefly described as follows. (1) Deposition of a thin layer of chromium (Cr) with a thickness of 600 nm on the surface of the LNOI by magnetron sputtering. (2) Patterning of Cr film using femtosecond laser ablation. (3) Removing the uncovered LNOI by chemo-mechanical polishing. (4) Chemical wet etching to remove the Cr mask. The pulse energy of the femtosecond laser was carefully adjusted to enable the complete removal of the Cr film without damaging the underneath LNOI because the damage threshold of the LNOI is significantly higher than Cr under the irradiation of femtosecond laser pulses. The details in the femtosecond laser ablation are provided herein. We used a femtosecond laser with a center wavelength of 1030 nm and a pulse width of 170 fs (PHAROS, LIGHT CONVERSION) for patterning the Cr film at the average power of 0.1 mW. A 100× objective lens (M Plan Apo NIR, Mitutoyo Corporation, NA0.7, Kawasaki, Kanagawa, Japan) was used to produce a tightly focused spot of ~1 μm in diameter.

In the CM polishing process using a wafer-polishing machine (NUIPOL802, Kejing, Inc.Hefei, China), the wedge angle can be controlled by changing the duration of the polishing process. More details of CM polishing can be found elsewhere [16,17].

## 3. Results and Discussion

**Figure 2:**
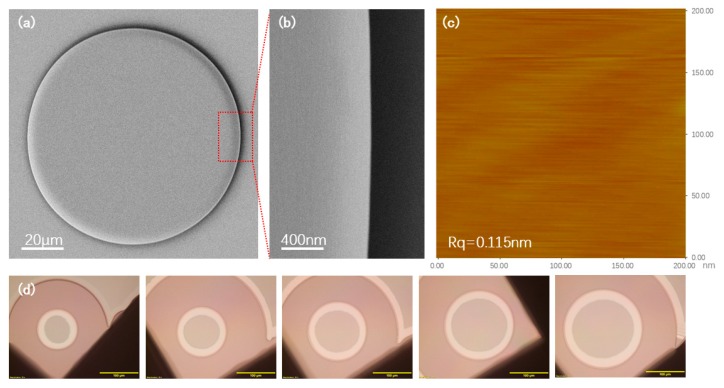
(**a**) Top view scanning electron microscope (SEM) image of a fabricated LN microdisk resonator. (**b**) Close up view SEM image of the area indicated by the red box in (**a**). (**c**) Atomic force microscope (AFM) image of the microdisk wedge. (**d**) Optical microscope image of the microdisk resonator with different diameters (55 μm, 85 μm, 105 μm, 135 μm, 155 μm, 185 μm, and 205 μm).

Figure 2a shows a LNOI microdisk fabricated using a SiO_2_ slurry with a particle size of ~20 nm. The close-up view of the area indicated by the red rectangular frame is shown in Figure 2b, presented a smooth surface morphology in the optical micrograph. Figure 2c presents the atomic force microscope (AFM) image of the polished surface by which an ultralow surface roughness of Rq ~0.115 nm could be determined. The surface roughness was significantly lower than that reported in our previous work (Rq ~0.452 nm) due to the fact that the slurry with a particle size of ~60 nm was used previously [16]. Actually, we systematically examined the surface roughness with the particle size and found that a particle size of ~20 nm could give us the optimum surface roughness and a reasonable polish duration. Figure 2d exhibits all the microresonators with different diameters ranging from 55 μm to 205 μm. All of the microresonators showed a highly reproducible smooth surface morphology.

**Figure 3:**
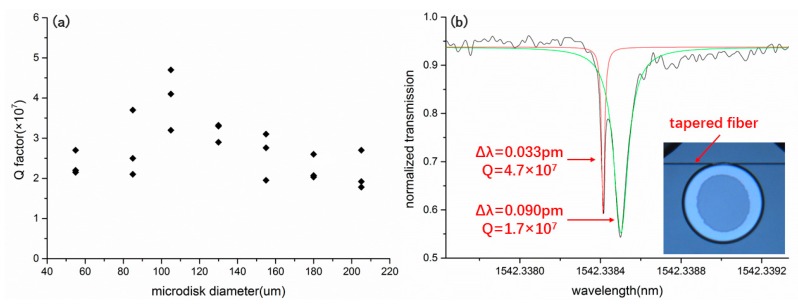
(**a**) Measured Q factors of the mcirodisks of different diameters. (**b**) The Lorentz fitting (red curve) of a splitting mode in the microdisk with a diameter of 105 μm revealed a Q factor of 4.7 × 10^7^.

With a surface roughness to the order of Ra ~0.115, the major source of optical loss in the microresonator is radiative bending loss, which can be reduced by increasing the diameters of the microdisks. To confirm this, we measured the Q factor of each microresonator in Figure 2d. The results are presented in Table 1 and Figure 3a. Indeed, it can be seen that the Q factor does undergo an increase for the microdisks with diameters of 55 μm, 85 μm, and 105 μm. However, after the Q factor reached its peak, which was 4.7 × 10^7^ obtained with the microdisk of a diameter of 105 μm, the Q factor decreased with the further increase of the diameter of the microdisk. It should be noted that to avoid the fluctuations of the measured Q factors resulting from the inconsistency in the fabrication process, we fabricated three samples for each diameter, and then made three measurements accordingly. The general trend in Figure 3a is therefore a reliable feature of the Q factor dependence on the microdisk diameter.

**Figure 4:**
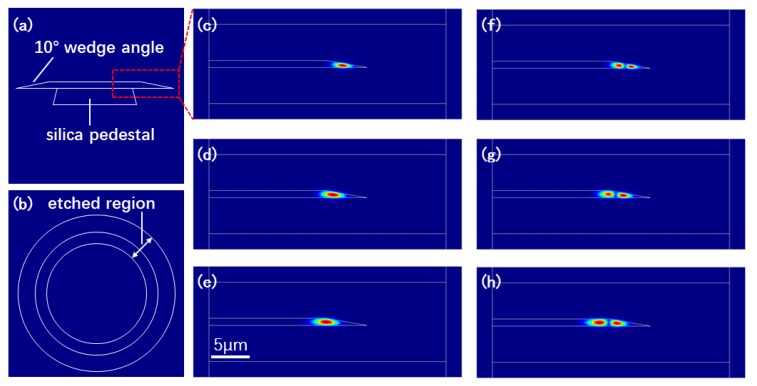
Side view (**a**) and top view (**b**) of the structure of the simulated microdisk resonator. Electric field of the fundamental modes in the microdisk resonator with a diameter of (**c**) 50 μm, (**d**) 100 μm, and (**e**) 200 μm. Electric field of second-order modes in the microdisk resonator with a diameter of (**f**) 50 μm, (**g**) 100 μm, and (**h**) 200 μm. The modes show a general feature where the mode size becomes larger with the increasing order of mode.

We attempted to understand the unexpected trend of the decreasing Q factor with the increasing microdisk diameter after the diameter of the microdisk reached 105 μm. To do so, we conducted simulations of the light fields (electric field) in the microdisks with different diameters but with the same wedge angle of 10°, and the thickness of the LN thin film was 900 nm. Note that we did not calculate the Q factors of the microdisks with different diameters as many key parameters (such as the exact diameter, thickness, and surface roundness of each microdisk) cannot be measured with absolute precision. The simulation results in Figure 4 revealed that with the increase in the diameter of the microdisk, the fundamental (Figure 4c–e) and high-order (Figure 4f–h) modes tended to penetrate more deeply toward the center of the microdisk. It is highly likely that the tail of the fundamental as well as high-order modes in the microdisks of large diameters may scratch the underneath pedestal supporting the freestanding microdisk, giving rise to a decrease in the Q factors. In our work, the diameter of the fused silica pedestal was controlled as ~20 μm less than that of the microdisk, otherwise the freestanding microdisk may collapse in the chemical wet etching to partially remove the fused silica. For this reason, some fused silica may remain unremoved on the backside of the microdisk during the short period of chemical wet etching, giving rise to a slight scattering loss and in turn, a slight decrease in the Q factors.

**Figure 5:**
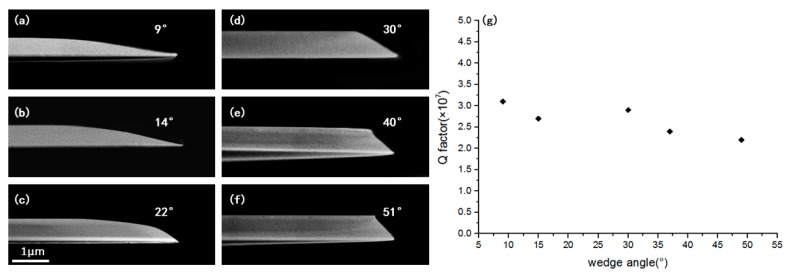
(**a**–**f**) Side view scanning electron microscope (SEM) image of the fabricated LN microdisks with different wedge angles of 9°, 14°, 22°, 30°, 40°, and 51°, respectively. (**g**) Q factors of the microdisks with different wedge angles.

We also investigated the dependence of the wedge angle of the microdisk on the polish duration. In general, the longer the polishing process, the larger the wedge angle of the microdisk. Figure 5a–f show the microdisks with a diameter of 85 μm obtained with polish durations of 12 min, 16 min, 18 min, 24 min, 30 min, 42 min, and 60 min, respectively. Accordingly, the wedge angles of the microdisks in Figure 5a–f were measured as 9°, 14°, 22°, 30°, 40°, and 51°, respectively. The measured Q factors of the microdisks with different wedge angles are provided in Figure 5g. The Q factors showed almost no dependence on the wedge angle, and were significantly higher than 1 × 10^7^ at all wedge angles.

**Figure 6:**
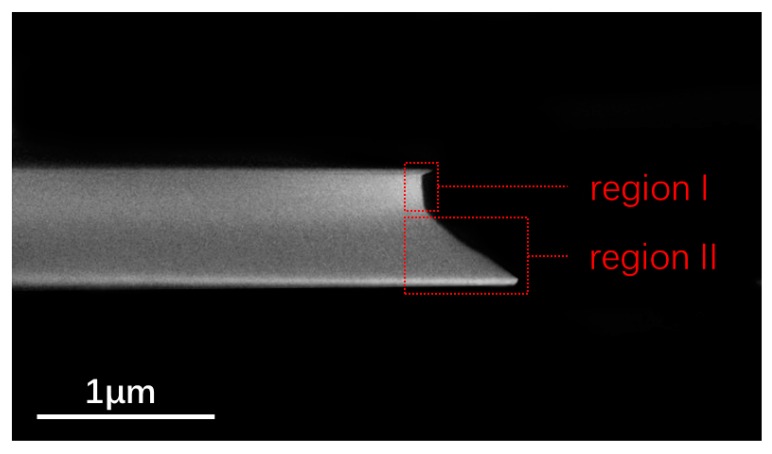
Side view scanning electron microscope (SEM) image of a fabricated LN microdisk resonator with a long polish duration of 90 min.

Finally, we noticed that from the side-view image in Figure 6, the microdisks of large wedge angles obtained with long polish durations appeared to be composed of a microdisk with a vertical sidewall (see region I in Figure 6) stacked on top of another microdisk with an extended wedge (see region II in Figure 6). The thicknesses of region I and II were measured as 280 nm and 420 nm, respectively. In general, this should be caused by the mechanical interaction of the LNOI microdisks with the polishing cloth, whereas understanding the underlying details requires further investigations in a systematic manner. Nevertheless, such complex geometry may provide innovative opportunities of controlling light fields in the LNOI microdisk resonators.

## 4. Conclusions

To conclude, we demonstrated the optimization of the Q factor and wedge angle of LNOI microdisk resonators fabricated by femtosecond laser assisted chemo-mechanical polish. We achieved, to the best of our knowledge, a record-high Q factor of 4.7 × 10^7^ by improving the surface smoothness and optimizing the diameter of the LNOI microdisk resonator. We showed that the wedge angle of the LNOI could be continuously tuned from 9° to 51° without spoiling the Q factor, which is critical for nonlinear optical applications as the dispersion curve in the microdisks is a function of the wedge angle. Thus, our results have important implications for applications ranging from classical and non-classical light sources to optical comb generation and optomechanics.

## Figures and Tables

**Table 1:** Q factors of the microdisks with different diameters.

Diameter	Q-Factors (Sample i)	Q-Factors (Sample ii)	Q-Factors (Sample iii)
55 μm	2.15 × 10^7^	2.26 × 10^7^	2.74 × 10^7^
85 μm	2.17 × 10^7^	2.51 × 10^7^	3.73 × 10^7^
105 μm	3.22 × 10^7^	4.16 × 10^7^	4.70 × 10^7^
130 μm	2.94 × 10^7^	3.32 × 10^7^	3.30 × 10^7^
155 μm	1.95 × 10^7^	2.76 × 10^7^	3.16 × 10^7^
180 μm	2.03 × 10^7^	2.07 × 10^7^	3.12 × 10^7^
205 μm	1.78 × 10^7^	1.92 × 10^7^	2.67 × 10^7^

## References

[1] Vahala K.J. (2003). Optical microcavities. Nature.

[2] Jiang X.F., Zou C.L., Wang L., Gong Q., Xiao Y.-F. (2016). Whispering-gallery microcavities with unidirectional laser emission. Laser Photonics Rev..

[3] Lin G., Chembo Y.K. (2019). Monolithic total internal reflection resonators for applications in photonics. Opt. Mater. X.

[4] Spillane S.M., Kippenberg T.J., Vahala K.J. (2002). Ultralow-threshold Raman laser using a spherical dielectric microcavity. Nature.

[5] Feng S., Lei T., Chen H., Luo X., Poon A.W. (2012). Silicon photonics: From a microresonator perspective. Laser Photonics Rev..

[6] Kippenberg T.J., Vahala K.J. (2008). Cavity optomechanics: Back-action at the mesoscale. Science.

[7] Foreman M.R., Swaim J.D., Vollmer F. (2015). Whispering gallery mode sensors. Adv. Opt. Photonics.

[8] Lin G., Coillet A., Chembo Y.K. (2017). Nonlinear photonics with high-Q whispering-gallery-mode resonators. Adv. Opt. Photonics.

[9] Spillane S.M., Kippenberg T.J., Painter O.J., Vahala K.J. (2003). Ideality in a fiber-taper-coupled microresonator system for application to cavity quantum electrodynamics. Phys. Rev. Lett..

[10] Lin J., Xu Y., Fang Z., Wang M., Song J., Wang N., Qiao L., Fang W., Cheng Y. (2015). Fabrication of high-Q lithium niobate microresonators using femtosecond laser micromachining. Sci. Rep..

[11] Wang J., Bo F., Wan S., Li W., Gao F., Li J., Zhang G., Xu J. (2015). High-Q lithium niobate microdisk resonators on a chip for efficient electro-optic modulation. Opt. Express.

[12] Liang H., Luo R., He Y., Jiang H., Lin Q. (2017). High-quality lithium niobate photonic crystal nanocavities. Optica.

[13] Boes A., Corcoran B., Chang L., Bowers J., Mitchell A. (2018). Status and potential of lithium niobate on insulator (LNOI) for photonic integrated circuits. Laser Photonics Rev..

[14] Lin J., Yao N., Hao Z., Zhang J., Mao W., Wang M., Chu W., Wu R., Fang Z., Qiao L. (2019). Broadband quasi-phase-matched harmonic generation in an on-chip monocrystalline lithium niobate microdisk resonator. Phys. Rev. Lett..

[15] Zheng Y., Fang Z., Liu S., Cheng Y., Chen X. (2019). High-Q exterior whispering-gallery modes in a double-layer crystalline microdisk resonator. Phys. Rev. Lett..

[16] Wu R., Zhang J., Yao N., Fang W., Qiao L., Chai Z., Lin J., Cheng Y. (2018). Lithium niobate micro-disk resonators of quality factors above 10^7^. Opt. Lett..

[17] Wu R., Wang M., Xu J., Qi J., Chu W., Fang Z., Zhang J., Zhou J., Qiao L., Chai Z. (2018). Long low-loss-lithium niobate on insulator waveguides with sub-nanometer surface roughness. Nanomaterials.

[18] Wang M., Wu R., Lin J., Zhang J., Fang Z., Chai Z., Cheng Y. (2019). Chemo-mechanical polish lithography: A pathway to low loss large-scale photonic integration on lithium niobate on insulator. Quantum Eng..

[19] Fang Z., Haque S., Lin J., Wu R., Zhang J., Wang M., Zhou J., Rafa M., Lu T., Cheng Y. (2019). Real-time electrical tuning of an optical spring on a monolithically integrated ultrahigh Q lithium nibote microresonator. Opt. Lett..

